# First Direct Evidence for Natal Wintering Ground Fidelity and Estimate of Juvenile Survival in the New Zealand Southern Right Whale *Eubalaena australis*

**DOI:** 10.1371/journal.pone.0146590

**Published:** 2016-01-11

**Authors:** E. L. Carroll, R. M. Fewster, S. J. Childerhouse, N. J. Patenaude, L. Boren, C. S. Baker

**Affiliations:** 1 School of Biological Sciences, University of Auckland, Auckland, New Zealand; 2 Scottish Oceans Institute, University of St Andrews, East Sands, St Andrews, Scotland; 3 Department of Statistics, University of Auckland, Auckland, New Zealand; 4 Blue Planet Marine, Kingston, Hobart, Tasmania, Australia; 5 Collégial International Sainte-Anne, Montréal, Québec, Canada; 6 New Zealand Department of Conservation, Wellington, New Zealand; 7 Marine Mammal Institute and Department of Fisheries and Wildlife, Hatfield Marine Science Center, Oregon State University, Newport, United States of America; University of British Columbia, CANADA

## Abstract

Juvenile survival and recruitment can be more sensitive to environmental, ecological and anthropogenic factors than adult survival, influencing population-level processes like recruitment and growth rate in long-lived, iteroparous species such as southern right whales. Conventionally, Southern right whales are individually identified using callosity patterns, which do not stabilise until 6–12 months, by which time the whale has left its natal wintering grounds. Here we use DNA profiling of skin biopsy samples to identify individual Southern right whales from year of birth and document their return to the species’ primary wintering ground in New Zealand waters, the Subantarctic Auckland Islands. We find evidence of natal fidelity to the New Zealand wintering ground by the recapture of 15 of 57 whales, first sampled in year of birth and available for subsequent recapture, during winter surveys to the Auckland Islands in 1995–1998 and 2006–2009. Four individuals were recaptured at the ages of 9 to 11, including two females first sampled as calves in 1998 and subsequently resampled as cows with calves in 2007. Using these capture-recapture records of known-age individuals, we estimate changes in survival with age using Cormack-Jolly-Seber models. Survival is modelled using discrete age classes and as a continuous function of age. Using a bootstrap method to account for uncertainty in model selection and fitting, we provide the first direct estimate of juvenile survival for this population. Our analyses indicate a high annual apparent survival for juveniles at between 0.87 (standard error (SE) 0.17, to age 1) and 0.95 (SE 0.05: ages 2–8). Individual identification by DNA profiling is an effective method for long-term demographic and genetic monitoring, particularly in animals that change identifiable features as they develop or experience tag loss over time.

## Introduction

Effective management of populations and species requires precise estimates of key demographic parameters, such as survival and rate of population change. While critical in the context of endangered and threatened species [1], such demographic parameters typically require long-term studies in which individuals are tracked through different life history stages. Such studies rely on individual identification using features that remain intact as the individual ages. Commonly used methods for identifying individuals include implanted or attached tags (e.g. PIT tagging [2]), genetic markers [3,4], assessment of phenotypes that differ between individuals such as acoustic calls [5,6] and photo-identification (photo-ID) of natural markings. Such natural markings can include part of the animal’s body, such as primate faces [7] and lion (*Panthera leo)* whisker patterns [8], or a composite of distinctive features, such as those used to identify Asian elephants (*Elephas maximus* [9]), tigers (*Panthera tigris* [10]), and marine species including various sharks and rays [11]. Computerised systems are increasingly used to recognise and match the phenotypes of individuals to create capture histories for use in demographic and ecological studies [12].

Many typical methods for identifying individuals may not be applicable to juveniles, particularly in large, long-lived species where the identifiable features of individuals can change with age. For example, tiger stripe patterns [10] and fluke patterns of humpback whale (*Megaptera novaeangliae* [13]) are not suitable for identifying young of the year and/or juveniles. In addition, cumulative marks may not be suitable for identification of juveniles. For example, bottlenose dolphins (*Tursiops* spp.) can be identified by scars, nicks or scratches on the dorsal fin [14] that are acquired by dolphins with age, therefore, adults are more likely to have such markings that make them individually identifiable.

There are exceptions where animals are identifiable from birth through natural markings (e.g., badgers *Meles meles*; [15], gray whales *Eschrichtius robustus*; [16]). However, it is more common that fewer juveniles than adults are individually identifiable, and those juveniles that are recognisable have markings that are distinctive or atypical [17], or have anomalies like albinism [18]. Furthermore, there can be heterogeneity between juveniles and adults in the proportion of individuals with distinctive markings, leading to juveniles being excluded from capture-recapture studies [17,19].

Monitoring juvenile movement and survival can be important to the management and conservation of species. Juvenile survival can show a greater sensitivity to environmental conditions than adult survival [20,21]. Furthermore, juveniles can make up a considerable proportion of a population [10], particularly in those populations that are growing [22]. Juvenile survival could therefore provide an early indicator of potential impacts on population growth rates and viability [23].

Demographic parameters for juveniles have been estimated indirectly using models such as life history or mortality tables [24], state space models [25] and matrix models [26]. Indirect methods do not track individual juveniles but instead deduce survival rates from the estimated numbers of animals in each age class and mean number of offspring per adult, relying on each individual’s progression through the demographic classes being largely constant and predictable. In cetaceans, other indirect methods for assessing calf and juvenile survival involve monitoring the association of offspring with photo-identifiable adult females, presumed to be their mother (e.g., [27]) and by evaluating the inter-calf interval in baleen whales. In the latter, a shorter than expected time interval between calving events implies that a calf has been lost and can be used as an indicator of calf mortality [28,29].

Direct methods, where individuals are marked and tracked, have the advantage of enabling fine-scale estimation such as differential survival between male and female juveniles, and are better able to accommodate intermittent field effort than indirect methods. Direct methods also allow juvenile survival to be estimated in species where juveniles take a long and variable time to reappear as adults in the sampled population, as is commonly the case for migratory whales and long-lived seabirds sampled at breeding grounds.

Here we focus on the use of genetic markers to monitor populations, which is becoming an increasingly important practice, particularly when there are conservation and management considerations [30,31]. Identification of individuals through genetic markers has several advantages over other methods, particularly for long-lived species. Unlike some forms of natural markings, genetic tags are stable and do not change over time. This makes them ideal to use for identifying individuals from birth.

Our study population is the New Zealand Southern right whale (*Eubalaena australis*), which was the subject of extensive commercial whaling that killed over 30,000 whales between 1827 and 1970 [32]. The contemporary New Zealand population is genetically distinct from Australian populations, and inhabits two wintering grounds where calving and breeding occur: the primary habitat in the New Zealand Subantarctic (centred at Port Ross, Auckland Islands) and the secondary habitat around mainland New Zealand (North and South Islands) [33].

Traditionally, photo-identification has been used to successfully identify non-calf right whales since the 1970s and focuses on callosity patterns found on the lip and rostrum, crenulations along the lower lip, and scars and unusual pigmentation on the head or body [34,35]. Such patterns do not typically stabilise until after 6 months of age, meaning dependent calves cannot normally be reliably identified from natural markings on their natal wintering ground [36]. Here we attempt to bridge this knowledge gap by identifying whales in their first 6 months of life using DNA profiles, comprising mitochondrial DNA haplotype, genetically identified sex and multilocus microsatellite genotype [37]. These data are used to investigate natal wintering ground fidelity for juvenile age classes and to provide the first estimate of juvenile survival in the New Zealand right whale population using a Cormack-Jolly-Seber (CJS) model [38].

## Methods

### Field and laboratory methods

**Fig 1:**
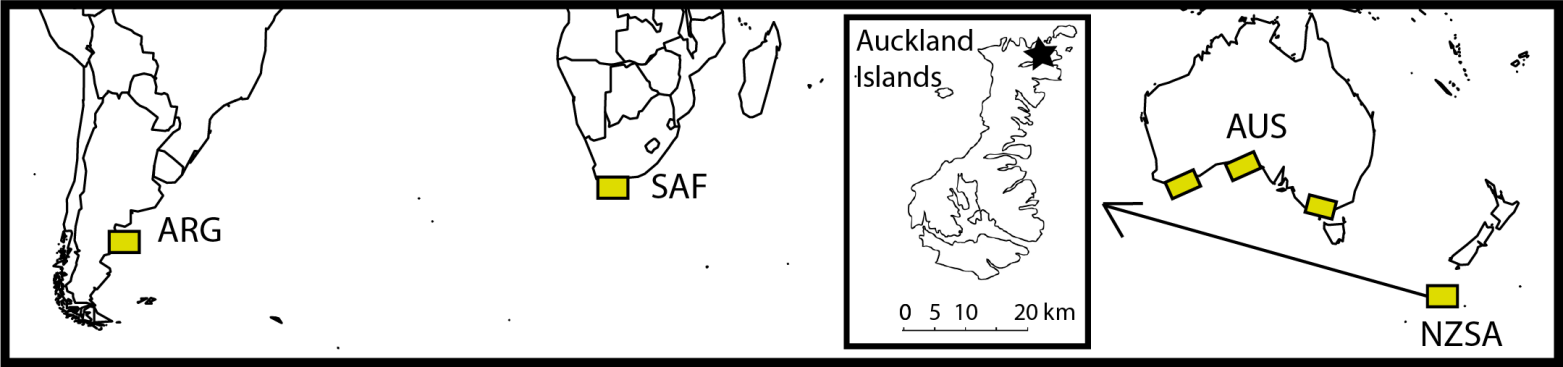
Location of primary Southern right whale breeding grounds and inset of study area at the Auckland Islands, New Zealand Subantarctic. Map of four of the primary Southern right whale wintering grounds including Península Valdés, Argentina (ARG), South Africa (SAF), Australia (AUS) and the New Zealand Subantarctic (NZSA). Inset shows a map of the Auckland Islands, with a star showing the location of Port Ross, the primary survey area.

Previous aerial and boat-based survey work indicates that the primary New Zealand wintering grounds of Southern right whales covers an area of approximately 20 km^2^, limited to the waters of Port Ross, in the Subantarctic Auckland Islands (50°32' S, 166°15' E) and nearby coastal waters (Fig 1) [39,40]. All age and demographic classes are present in this wintering ground, meaning samples taken here are likely to be representative of the overall population [41,42]. Surveys were designed to coincide with peak abundance of Southern right whales in mid-July to early August [41,43] and were conducted during a total of 8 austral winters from 1995–1998 and 2006–2009.

As previously described [39], surveys were conducted from small vessels (4.6–5.2m). To avoid biases in sampling, search effort was distributed approximately evenly across the study area and attempts were made to approach every group sighted as described by Carroll et al. [44]. Skin biopsy samples were collected using small, stainless steel biopsy darts deployed from a crossbow in 1995–1998 [45] or a modified veterinary capture rifle in 2006–2009 [46] as previously described [33,47]. Biopsy samples were collected during the 2006–2009 field seasons under Department of Conservation (DOC) Marine Mammal Research permit and University of Auckland Animal Ethics Committee approved protocol to C. S. Baker. Biopsy samples were collected during the 1995–1998 field seasons under permit from the New Zealand DOC to C. S. Baker and N. Gales and University of Auckland Animal Ethics Committee approved protocol to C. S. Baker. Skin biopsy samples were preserved in 70% ethanol on location and transferred to the University of Auckland for storage at -20°C.

In the field, sampled whales were classified into two age groups based on their body length: non-calf or calf. The latter was defined as a whale whose portion of body visible at the surface was less than half of the length of an accompanying adult, and such an animal was assumed to be in its first year of life (age 0). A whale in close association with a calf was assumed to be its mother and was noted as a cow. In the subsequent modeling exercises, we define juveniles to be whales aged between 1 and 8 years. Age assignments were based solely on the progression of known-aged whales first captured as calves: in the field juveniles and adults are not consistently distinguishable. As reproductive maturity in the Southern right whale occurs by 9 years of age in females, we considered 8 years as the conservative maximum estimate of juvenile age [48–50].

DNA was extracted using standard proteinase K digestion and phenol/chloroform methods [51], as modified for small samples by Baker et al. [52]. DNA profiles, comprising genetically identified sex, mitochondrial control region (mtDNA) haplotypes and multi-locus genotypes, were constructed following protocols previously described [44]. Briefly, the sex of the sampled whale was identified by amplification of the male-specific SRY gene, multiplexed with an amplification of the ZFY/ZRX region as positive control [53,54]. Sequencing of the mitochondrial control region (500 bp) was conducted using the primers dlp1.5 [55] and tphe [33], the former modified with a 5’-M13 primer extension to facilitate subsequent sequencing reactions. The subsequent PCR product (~950 bp) was purified for sequencing with ExoSAP-IT (USB, Cleveland, Ohio, USA) and sequenced using BigDye Dye Terminator Chemistry (Applied Biosystems, Foster City, California, USA) on an ABI 3730 or ABI 3130 (Applied Biosystems). Sequences were aligned and edited in Sequencher v4.2 (Gene Codes Corporation, Ann Arbor, Michigan, USA) or Geneious [56], and haplotypes were identified from a 500 bp consensus region using haplotype codes established by Carroll et al. [33].

We undertook microsatellite genotyping using thirteen microsatellite loci (EV1, EV37, and EV14 [57]; GATA28 and GATA98 [58]; RW18, RW31, RW410, and RW48 [59]; GT23 [60]; TR3G1, TR3G2 and TR3F4 [61]), each amplified in individual 10 μL PCR reactions as previously described by Carroll et al. [33]. Each 96-well tray included a set of seven standard samples as an internal control to ensure consistent allele sizing and a negative control to detect contamination. Amplicons from 4–6 loci were co-loaded for capillary electrophoresis with an ABI 3730 or an ABI 3130. Alleles were sized with Genemapper v 4.0 (Applied Biosystems) and all automated calling was confirmed by visual inspection.

Identification of matching genotypes, i.e., recaptures, was conducted as described in Carroll et al. [33]. Briefly, a quality control measure of amplifying at a minimum of 9 loci was employed and matching genotypes were identified using CERVUS v3.0 [62]. As a precaution against false exclusion due to allelic dropout and other genotyping errors [63,64], the initial comparison allowed for mismatches at up to three loci.

We make the assumption that any calf captured at the Auckland Islands was born there, given the remote location and comparatively poor swimming capacity of right whale calves in their first few weeks of life [65]. We consider the calf to be recaptured in the Auckland Islands if it was sampled in a subsequent year.

### Evaluating model assumptions

To estimate survival, we used an open capture-recapture model, the Cormack-Jolly-Seber (CJS) model. This capture-recapture model has several underlying assumptions (for a review see [19]), including (1) marks are permanent and can be correctly identified by researchers; (2) there is no behavioral response to capture; (3) there are equal survival and capture probabilities within demographic classes and (4) the population has geographic closure [66].

Individuals were identified using DNA profiles, which included a multilocus microsatellite genotype comprising an average of 12 loci per individual [37]. As previously reported, the least variable 11 loci provided a probability of identity (P_ID_), or the chance that two individuals will have the same genotype by chance, of 7.9 x 10^−14^ [67], and a probability of identity for siblings, or the probability that two closely related individuals will have the same genotype by chance, of 1.73 x 10^−5^ [68]. Given these low probabilities, we considered that the suite of microsatellite loci could be used to confidently differentiate between even closely related individuals in a population estimated to have a superpopulation size of 2169 whales over the period 1995–2009 [37]. The low per allele error rate of 0.61% [37] and relaxed matching process means that it is unlikely that genotyping error would lead to a missed recapture event.

To evaluate assumption (2), behavioral response to capture, and to test for heterogeneity in survival probability between individuals, most commonly attributed to transiency, we used the program U-CARE [69]. To evaluate assumption (3), we tested whether there were differences in the recapture rates between males and females first sampled as calves and between the two sampling periods with Fisher’s exact test. Regarding assumption (4), the same wintering area, the Port Ross region of the Auckland Islands, was surveyed in each of the 8 expeditions [37].

The information from the recapture of adult individuals over time has been used to estimate the abundance of the population in both 1998 and 2009 [37,44], and to track movements of individuals between mainland New Zealand and the New Zealand Subantarctic [70]. However, existing analyses of recapture data, while being sex-specific, have not been linked to age classes. Furthermore, as calf survival is thought to be lower than that of adults in the Southern right whale [71], this age class was excluded from all previous analyses. In this study, all modeling was done using known-age whales that were first sampled in year of birth, i.e. first sampled as calves.

### Mark recapture modeling

We used the CJS model, coded and implemented in R [72], to estimate the annual survival probability for calves, covering the year from age 0 to age 1, and for subsequent juvenile and adult age classes. The annual probability of apparent survival (Φ) was modelled as age-invariant Φ(.), or allowed to differ in the first year of life but remain constant thereafter (Φ_A2_), following previous studies in other right whale populations which suggested that survival in the first year of life might be lower than that in subsequent years [71].

In addition, we considered a model denoted Φ_(curve)_ for continuous change in survival probability with age, in contrast with discrete age categories. We developed this model because Southern right whale calves grow rapidly in the first year of life [73] and the closely related North Atlantic right whale is estimated to reach 75% of its adult size by age one due to high maternal investment [74]. We postulate that survival may increase through the juvenile years due to the increased protection that size brings from predators such as killer whales and large sharks, eventually reaching a plateau at the adult survival level [48–50]. We used the following family of curves to model the relationship between age and apparent survival:

$$\Phi \left(a\right)\;=\;\kappa \left\{1-\text{exp}\left(-\frac{a}{\sigma }\right)\right\}$$

where κ and σ are parameters to be estimated, and Φ(*a*) denotes the probability of apparent survival from age *a* − 1 years to age *a*. Parameter κ (0 < κ < 1) specifies the final adult survival rate at the plateau, and parameter σ (σ > 0) determines how quickly the survival curve reaches the adult level, with small values of σ indicating that juveniles quickly attain adult survival probabilities.

For each of the standard CJS and Φ_(curve)_ models, we explored models with constant capture probability (*p*(.)), and capture probability that varied with year or capture occasion (*p*(*t*)), and according to survey period (1995–1998 and 2006–2009), denoted *p*(90s,00s). In total we fitted nine models, combining each of three choices for survival (Φ(.), Φ_A2_, and Φ_(curve)_) with each of three choices for capture probability (*p*(.), *p*(*t*), and *p*(90s,00s)). Model-fitting was conducted by maximum likelihood, and we checked that our R results were identical to those from program MARK [75] for the six models with discrete age classes that could be constructed in program MARK.

In view of the small sample size of 66 capture histories, we used a bootstrap analysis [76] to construct confidence intervals. We randomly sampled the 66 capture histories with replacement to create 1000 bootstrapped datasets, each containing 66 capture histories and each representing animals first captured as calves. The model of interest was fitted to each bootstrapped dataset, generating 1000 estimates of each parameter including the survival probabilities Φ(*a*) for ages *a* = 1, 2, …14, with age 14 being the oldest possible age of animals in the study from 1995–2009. Bootstrapped 95% confidence intervals were taken from the 2.5% to the 97.5% quantiles of the 1000 estimates. We checked the bootstrap confidence intervals against analytic confidence interval computations, and found that in most cases the bootstrap confidence intervals were slightly wider. We used the bootstrap approach because it does not rely upon the large sample sizes that are assumed for analytic calculations, and because it is able to generate meaningful estimates of uncertainty in cases where analytic calculations fail.

The Akaike Information Criterion (AIC [77]), corrected for small sample sizes (AICc [78]), was used to assess support for each model [79]. As several models were closely ranked by AICc, we conducted an overall bootstrap analysis to give a single model-averaged estimate and confidence intervals for each survival probability, incorporating model-selection uncertainty. This approach is recommended by Buckland et al [80,81]. We generated 1000 bootstrapped datasets as before. To each bootstrapped dataset, we fitted all nine models, selected the best model based on AICc, and used the selected model to derive the age-based estimates of survival corresponding to that bootstrap iteration, namely Φ(*a*) for ages *a* = 1, 2, …14. The model-averaged estimate and standard error (SE) of each Φ(*a*) is given by the mean and standard deviation of the 1000 bootstrapped estimates, and the model-averaged 95% confidence interval for Φ(*a*) is taken from the 2.5% to the 97.5% quantiles of the 1000 estimates.

## Results

### Sample sizes and between-year recaptures

Over the 8 years of field work (1995–1998 and 2006–2009), 66 samples from calves produced DNA profiles that passed the quality control criterion of amplifying at a minimum of 9 of 13 loci. Of these, 34 were male and 32 were female; sample size and recaptures by years are shown in Table 1.

**Table 1:** The number of between-year recaptures of Southern right whales first sampled as dependent calves during austral winter field surveys at the Auckland Islands from 1995–1998 and 2006–2009.

		Year of initial capture							
A. Males		1995	1996	1997	1998	2006	2007	2008	2009
	N_M_	2	2	1	1	7	9	6	6
	1996	0							
Year of	1997	0	0						
recapture	1998	0	0	0					
	2006	0	0	0	0				
	2007	0	1	0	0	3			
	2008	0	0	0	0	1	0		
	2009	0	0	0	0	2	1	0	
		Year of initial capture							
B. Females		1995	1996	1997	1998	2006	2007	2008	2009
	N_F_	2	0	1	3	1	11	11	3
	1996	1							
Year of	1997	0	0						
recapture	1998	1	1	0					
	2006	1	1	0	1				
	2007	0	0	0	2	0			
	2008	0	0	0	0	0	2		
	2009	0	0	0	0	0	2	1	

A. The number of male calves identified using microsatellite genotype data (N_M_) and the number of males recaptured between years. B. The number of female calves identified using microsatellite genotypes (N_F_) and the number of females recaptured between years. One individual is recaptured in more than one year and is counted as multiple recaptures in the Table.

During the 1995–1998 field survey period, 12 calves were sampled: 6 females and 6 males. Of these, 3 females and 1 male were recaptured as presumed adults in the 2006–2009 field surveys. Two female calves that were sampled in 1998 were subsequently recaptured as cows with calves in 2007. As female maturity in right whales is estimated at between 7 and 9 years [50,71,82], it is likely these records represent the first parturition for these females. Another female, first sampled as a calf in 1995, was subsequently recaptured in 1996, 1998 and 2006: each time without a calf. The male calf, first captured in 1996, was recaptured as a presumed adult, 11 years later, in 2007.

There were 54 dependent calves sampled during the 2006–2009 survey period: 26 females and 28 were males. Of these calves, 9 were sampled in 2009 and not available for subsequent recapture. Of the 45 available for recapture in at least one year, 9 were sampled in two years (20%; 4 males and 5 females), and one male was sampled in three years (2%). The P_ID_ of these matches was between 5.76 x 10^−23^ and 4.70 x 10^−14^ (average 3.96 x 10^−15^), based on between 8 and 13 loci (average of 11 loci, S1 Table shows matching genotypes).

### Estimates of calf survival

The U-CARE tests indicated no evidence of violations in the assumptions of mark-recapture models: both Test 3.SR (two sided test, p = 0.73), which tests for equal survival probability, and Test 2.CT (two sided test, p = 0.64), which tests for behavioural response to capture, were non-significant. There was no evidence of heterogeneity in capture probability linked to sex in the juvenile dataset. The recapture rate between females (5/28) and males (8/29) was not significantly different (2009 samples excluded as they could not be recaptured; Fisher’s exact test, p = 0.53). Similarly, the recapture rate of individuals sampled in the differing sampling periods was not significantly different (1995–1998: 4/12 and 2006–2009: 10/45, Fisher’s exact test p = 0.46). Therefore data were pooled for subsequent analyses.

**Fig 2:**
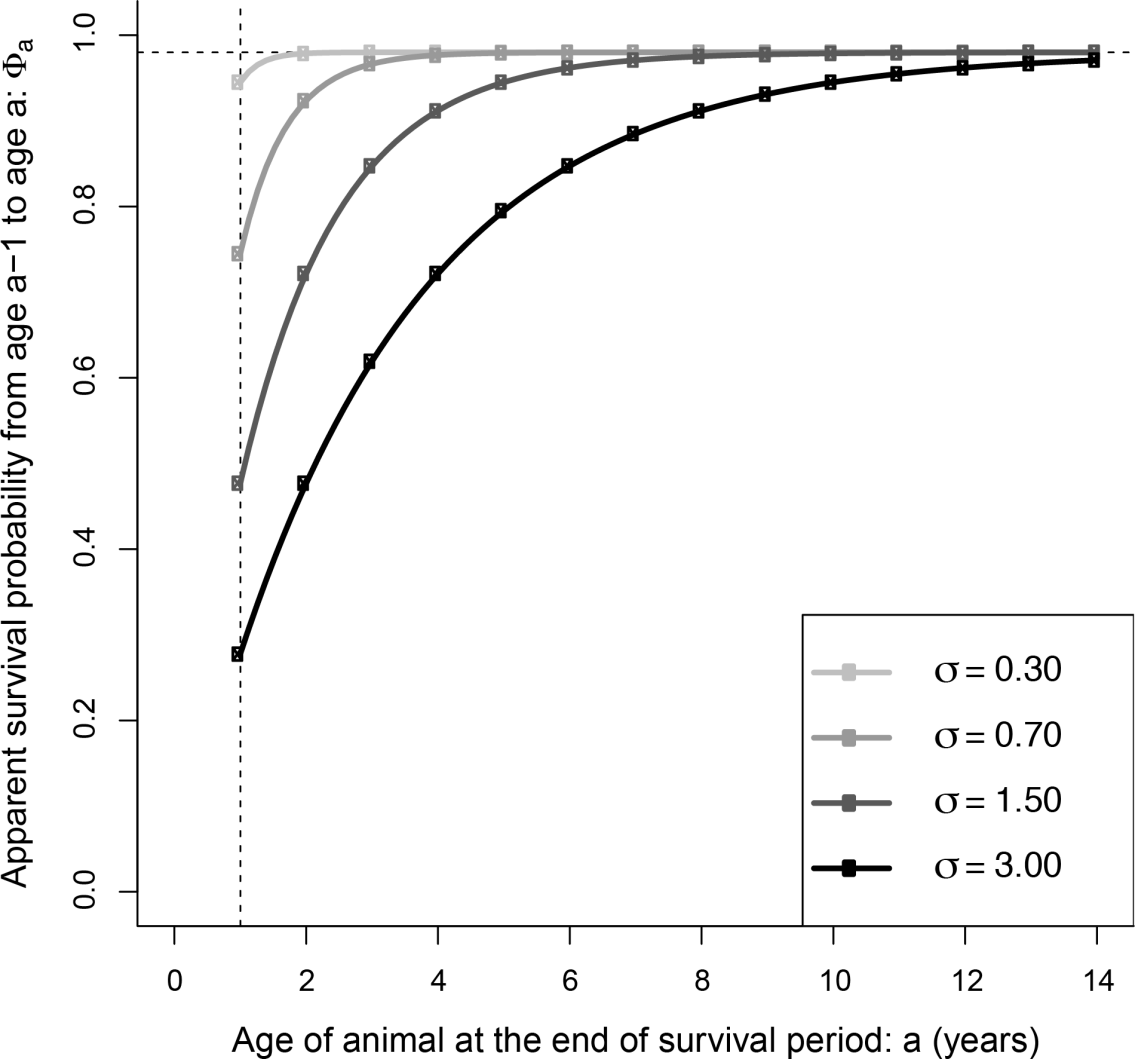
Family of curves used to model annual apparent survival as a function of age in the Φ(curve) models. The models follow the equations, $\Phi \left(a\right)\;=\;\kappa \left\{1-\text{exp}\left(-\frac{a}{\sigma }\right)\right\}$, where κ represents adult survival (κ = 0.98 in curves shown here) and σ controls the speed at which adult survival is attained. Curves where σ<1.0 fit the data best, indicating survival plateaued at a young age, compared with σ = 3.0 where survival plateaus at around age 8.

Based on AICc, the Φ(.)*p*(.) CJS model was most highly ranked and produced an estimate of apparent annual survival of 0.96 for all ages (95% confidence intervals 0.85, 1.00: Table 2). However, several models were closely ranked (ΔAICc 1.75–2.16), including models that permit survival to vary with age. The fit of these models indicated that only the first two years of life has a decrease in survival probability relative to adults or other juvenile classes: the Φ_(curve)_ model had the flexibility to allow survival to take several years to plateau at the adult level, but it estimated the plateau to be reached by age three (see reference curves in Fig 2). This was also borne out by the bootstrap analysis, which estimated apparent annual survival to age one to be 0.87 (SE 0.17), increasing to 0.95 (SE 0.05) in subsequent years (Table 3). However, most of the models (80%) selected by the 1000 bootstrap replicates had age-invariant survival, indicating little support for a lower survival in the first year (Table 2).

**Table 2:** Cormack-Jolly-Seber model estimates of apparent annual survival and 95% confidence intervals (95% CI) for juvenile New Zealand Southern right whales.

Model	ΔAICc	N_boot_	Φ(.)	Φ_A1_	Φ_A2+_	Φ_(curve)A1_	Φ_(curve)A2_	Φ_(curve)A3+_	σ	κ
			(95% CI)	(95% CI)	(95% CI)	(95% CI)	(95% CI)	(95% CI)		
Φ(.)*p*(.)	0.00	434	0.96							
			(0.85, 1.00)							
Φ_A2_*p*(.)	1.75	68		0.68	0.99					
				(0.38, 1.00)	(0.86, 1.00)					
Φ_(curve)_*p*(.)	1.86	3				0.76	0.93	0.97	0.69	0.99
						(0.53, 0.99)	(0.76, 1.00)	(0.84, 1.00)		
Φ(.)*p*(90s,00s)	2.16	162	0.96							
			(0.83, 1.00)							
Φ_A2_*p*(90s00s)	3.97	18		0.63	1.00					
				(0.34, 1.00)	(0.86, 1.00)					
Φ_(curve)_*p*(90s00s)	4.12	4				0.74	0.93	0.98	0.73	0.99
						(0.55, 0.98)	(0.78, 1.00)	(0.83, 1.00)		
Φ(.)*p*(t)	4.88	195	0.94							
			(0.82, 1.00)							
Φ_A2_*p*(t)	6.95	66		0.55	1.00					
				(0.34, 1.00)	(0.85, 1.00)					
Φ_(curve)_*p*(t)	7.47	50				0.76	0.93	0.96	0.64	0.97
						(0.54, 0.96)	(0.76, 0.99)	(0.81, 1.00)		

Estimates are based on Southern right whales first sampled as calves at the Auckland Islands, New Zealand during two sets of winter field surveys; 1995–1998 and 2006–2009, ranked by AICc, with N_boot_: the number of times the model was selected as the best fitting model by AICc during the bootstrap analysis; Φ(.): apparent survival is age-invariant; Φ_A2:_ apparent survival varies between first and subsequent years of life; Φ_(curve)_ apparent survival is modelled as a function of age where $\Phi \left(a\right)\;=\;\kappa \left\{1-\text{exp}\left(-\frac{a}{\sigma }\right)\right\}$, with estimates for the first (Φ_(curve)A1_), second (Φ_(curve)A2_) and third and subsequent years of life (Φ_(curve)A3+_) shown; *p*(.): capture probability is time-invariant; *p*(*t*): capture probability varies with capture occasion; *p*(90s00s): capture probability varies with survey period; estimates of σ and κ are also given for the Φ_(curve)_ models.

**Table 3:** Model averaged estimates of apparent annual survival of Southern right whales from New Zealand.

Parameter	Estimate	Standard error	Lower 95% CI	Upper 95% CI
ΦA_1_	0.87	0.17	0.38	1.00
ΦA_2_	0.95	0.05	0.81	1.00
ΦA_3_	0.95	0.05	0.82	1.00
ΦA_4_	0.95	0.05	0.82	1.00
ΦA_5_	0.95	0.05	0.82	1.00
ΦA_6_	0.95	0.05	0.82	1.00
ΦA_7_	0.95	0.05	0.82	1.00
ΦA_8_	0.95	0.05	0.82	1.00
*p*_1996_	0.20	0.20	0.00	1.00
*p*_1997_	0.10	0.10	0.00	0.32
*p*_1998_	0.16	0.14	0.00	0.58
*p*_2006_	0.16	0.10	0.00	0.41
*p*_2007_	0.29	0.22	0.07	0.81
*p*_2008_	0.13	0.07	0.02	0.29
*p*_2009_	0.14	0.07	0.04	0.31

Survival (Φ) is given for ages 1 to age 8, denoted A_1_-A_8_, in addition to capture probability by year, denoted p_year_. Lower and upper 95% confidence intervals (95% CI) are shown for each estimate.

It is notable that there is a hierarchical nature to the model ranking, with the models with constant capture probability ranking highest, followed by those models with survey-dependent capture probability and lastly models with time-dependent capture probabilities (Table 2). Within each capture probability configuration, the time-invariant survival model ranks first, the first-year survival model ranks second and the curve-based model ranks third.

## Discussion

Here we have successfully used genetic tags in the form of DNA profiles to identify recaptures of juvenile Southern right whales first caught as calves, for the purpose of investigating juvenile survival and fidelity. In doing so, we highlight the use of genetic monitoring and maintaining a catalogue of DNA profiles as a method to address a gap in methodology: a method to reliably identify juveniles in species where natural markings are not sufficient to identify young individuals or where animals change as they develop.

### Direct evidence of fidelity to natal wintering grounds in New Zealand Southern right whales

The recapture of male and female calves first sampled in the 1990s returning to the New Zealand Subantarctic up to 11 years later provides first direct evidence of long-term, maternally-directed fidelity to this wintering ground. This is consistent with long-term studies in conspecific populations that are based on the resighting of the small proportion of calves that are photo-identifiable through white dorsal pigmentation anomalies. For example, Best et al. [83] estimated that at least 93.4% of female calves born in the South African wintering ground return there to have their first calf. In Península Valdés, Argentina, 54 of 92 (59%) whales first identified as calves from 1971–1989 were resighted at the wintering ground by 1990 [84]. This study also suggested whales were more likely to be resighted when they were juveniles aged 1–4 (34% of possible resightings were observed) than as adults aged 4+ (23% of possible resightings observed). A comparison between the New Zealand and Argentinean wintering grounds is not possible as the eight-year gap between the two sets of consecutive surveys limited the opportunity to recapture calves for three years after birth. The only years where this was possible was in 1995 and 2006; of the 13 calves captured in these years, 5 (38%) were recaptured in the subsequent three years.

### Estimating juvenile survival in New Zealand Southern right whales

Here we provide the first estimate of juvenile survival for the New Zealand population of Southern right whales. Using known-age whales and a flexible modelling framework, we were able to investigate changes in survival with age using discrete and continuous age classes. Overall, our bootstrapped results suggest apparent annual juvenile survival (ages 2 to 8) is high at 0.95 (SE 0.05), with only weak evidence for a decrease in apparent survival in the first year of life to 0.87 (SE 0.17).

The sample size used in this study was small (n = 66, 57 of which were available for recapture), a problem that is difficult to avoid in a population that is recovering from exploitation. This small sample size has implications for our ability to test for violations in model assumptions and impacts the precision of our estimates. For example, we have found low support for a decrease in survival in the first year of life in our model-average exercise: only 20% of bootstrap replicates selected a model that supported such a decrease. The small sample size means that we might not have had the power to detect such a pattern, which is a common mammalian trait [85]. Our ability to detect a difference in the recapture rate between males and females is also limited by sample size. However, while reproductively mature females show a clear pattern in recapture linked to reproductive cycle [37], there is no reason to expect such a pronounced difference in the pattern or rate of recaptures in non-reproductive females and males.

Finally, we did not investigate or account for individual-level heterogeneity. This could be investigated using a random effects model (e.g., [86]), but such models require larger sample sizes than we have available. Survival estimates from the CJS model are relatively robust to individual-level heterogeneity in capture probabilities [19,87]. Another potential problem is that we are estimating apparent survival, a composite of true survival and fidelity. Although we have presented evidence of long-term fidelity in the New Zealand population, there can be plasticity in this philopatric behaviour [88]. Therefore, we could be under-estimating the survival rate if fidelity is lower than expected and there is permanent emigration. Given the estimates of survival are high, this does not appear to be a major concern.

Despite our limitations, our estimates and precision are comparable to those from other studies on baleen whales, including Southern right whales. For example, first year female survival of Southern right whale was estimated at between 0.713 (95% confidence intervals 0.529, 0.896) and 0.956 (SE 0.051), using a multi-state matrix model that integrates capture histories of reproductively mature females captured at the South African wintering ground [48,71]. Female survival for ages 2+ was estimated to be 0.988 (SE 0.001) using the same model [48]. Estimates for the Península Valdés population using a similar modelling procedure suggested survival rate to reproductive maturity was 0.92 (SE 0.11) for females, implying an average annual mortality rate of calves and juveniles of about 0.01 (SE 0.01) [82].

Although estimates of juvenile survival in cetacean species are rare, our estimate of apparent survival in New Zealand right whales is higher than those available for gray whales: a study based on the capture-recapture of 129 photo-identified western gray whales (*Eschrichtius robustus*), which used Pollock's robust design model, estimated first year calf survival to be 0.701 (SE 0.0994, 95% confidence intervals 0.492, 0.850) and non-calf survival to be 0.951 (SE 0.0135, 95% confidence intervals 0.917, 0.972) [16]. Gabriele et al. [89] used an indirect method to calculate calf mortality in humpback whales by estimating the proportion of mothers identified with a calf in Hawai'i but without a calf in Alaska. This gave a point estimate of calf mortality of between 0.15 (95% confidence intervals 0.032, 0.378) and 0.241 (95% confidence intervals 0.103, 0.434), depending on whether all calf absences were attributed to mortality or a portion were attributed to weaning. Using an indirect method similar to that of Brandão et al. [48] that incorporates the calving intervals of humpback whales from the Gulf of Maine into a maximum likelihood framework, Barlow & Clapham [90] estimated humpback whale calf survival to be 0.875 (SE = 0.047). In an example from the small cetacean literature concerning bottlenose dolphins in Shark Bay, Australia, Mann et al. [27] estimated calf mortality by monitoring the association of offspring with photo-identifiable adult females, presumed to be their mother. In this study, 44% of bottlenose dolphin calves died by age 3, and a study of bottlenose dolphins at Doubtful Sounds, New Zealand, using similar methodology found calf survival to age 3 was 40% [91].

The high survival rate presented here and in the literature generally for Southern right whales may be due to analytical or biological effects. There is evidence to suggest that in the closely related species, the North Atlantic right whale, calves have a higher level of maternal investment than other baleen whale species, such as prolonged lactation period during which the calf can reach 75% of its expected adult length [74]. This optimised size at weaning may facilitate a higher juvenile survival in North Atlantic right whales, and the related Southern right whale, compared with other cetacean species.

### Genotyping error and individual identification

Skin biopsy samples generally produce DNA of quality superior to that of typical ‘non-invasive’ sources of DNA. This is shown by the low error rate of the overall DNA profile dataset used in this study (0.61% per allele). This is similar to error rates estimated in studies that used tissue samples (0.8% for tissue [92],1.3% per locus and 0.8% per allele [93]). Studies which use non-invasively collected samples often have higher estimates of error, for example, error rates of 2.0%–11.3% and 2.55%–18.7% have been reported for studies using DNA extracted from faeces and hair, respectively [92,94,95]. We also attempted to minimise genotyping error from the start of the study by following published recommendations e.g., assessing DNA quantity and quality and ensuring samples are tracked accurately through the genotyping process [92,96].

However, even low error rates across large datasets, particularly those based on samples of varying quality, mean that genotyping error needs to be assessed and accounted for. Here we did this by estimating the genotype error rate and by matching samples in a relaxed framework. By identifying soft matches–those that match at multiple loci but that mismatch at up to three loci–we identify recaptures while allowing for genotyping error in the dataset. A study of misidentification error based on the adult captures from these field seasons, which were genotyped and matched by the same protocols, failed to find any evidence of matching errors [97].

Linking genetic samples to independent datasets of other tag types, such as natural markings or attached tags, has the potential to be an even more powerful method. Indeed, combining different tag types allows for animals to be tracked by different research groups over space and time, and is known to be statistically more informative than using a single tag [98,99].

### Conclusion

The use of genetic tags allows individuals to be identified from birth, and is suitable for use with intermittent field effort, as was the case in this study. Genetic tagging is just one of many and varied methods to investigate fidelity and survival in calf and juvenile age classes. In the Southern right whale alone, there have been several indirect methods used, in addition to our direct method based on genetic tagging. This is an important topic as environmental conditions at high-latitude feeding grounds are hypothesised to affect population dynamics through reproductive success and juvenile survival in the Southern right whale [100,101]. The use of genetic monitoring means that there is no tag loss and all individuals can be identified from birth. We suggest integrating this method into the toolkit used to monitor the New Zealand Southern right whale and other baleen whale populations. In addition, increased sampling at both winter breeding and summer feeding grounds would enable more precise estimation of survival, and might enable application of covariate models to investigate impacts of environmental variables and specific feeding grounds.

## Supporting Information

S1 Appendix Capture histories for southern right whales first captured as calves during field trips to the Auckland Islands, New Zealand Subantarctic in 1995–1998 and 2006–2009. (INP)

S1 Table DNA profiles for southern right whales first captured as calves and recaptured in at least one subsequent year, during field trips to the Auckland Islands, New Zealand Subantarctic in 1995–1998 and 2006–2009. (DOCX)
