# Supplementary material for: First Direct Evidence for Natal Wintering Ground Fidelity and Estimate of Juvenile Survival in the New Zealand Southern Right Whale Eubalaena australis
Source: PLoS One. 2016 Jan 11;11(1):e0146590. doi: 10.1371/journal.pone.0146590 (PMC4709107; doi:10.1371/journal.pone.0146590)
Supplement: S1 Table — (DOCX) [file pone.0146590.s002.docx]

S1 Table: DNA profiles for southern right whales first captured as calves and recaptured in at least one subsequent year, during field trips to the Auckland Islands, NZ subantarctic in 1995-1998 and 2006-2009. DNA profiles comprise genetically identified sex (Sex), mitochondrial control region haplotype (mtDNA: haplotype code as described in Carroll *et al.* 2011*b*) and multilocus genotype (EV1 – TR3G2). The demographic category (DC), number of matching loci (N_M_), number of mismatching loci (N_MM_) and number of loci each sample is amplified at (N_loci_) are also reported. Missing data at a locus are denoted by 0.

| **Sample Name** | **Year** | **N_M_** | **N_MM_** | **mtDNA** | **sex** | **DC** | **EV1** | | **EV14** | | **EV37** | | **GATA28** | | **GATA98** | | **GT23** | |
| --- | --- | --- | --- | --- | --- | --- | --- | --- | --- | --- | --- | --- | --- | --- | --- | --- | --- | --- |
| Eau95AI017 | 1995 | 8 | 0 | BakHapA | F | calf | 128 | 136 | 133 | 137 | 197 | 207 | 166 | 178 | 108 | 116 | 112 | 114 |
| Eau96AI017 | 1996 | to |  | BakHapA | F | non-calf | 128 | 136 | 133 | 137 | 197 | 207 | 166 | 178 | 108 | 116 | 112 | 114 |
| Eau98AI035 | 1997 | 13 |  | BakHapA | F | non-calf | 128 | 136 | 0 | 0 | 0 | 0 | 166 | 178 | 108 | 116 | 112 | 114 |
| Eau06AI039 | 1998 |  | 0 | BakHapA | F | non-calf | 128 | 136 | 133 | 137 | 197 | 207 | 166 | 178 | 108 | 116 | 112 | 114 |
|  |  |  |  |  |  |  |  |  |  |  |  |  |  |  |  |  |  |  |
| Eau98AI106 | 1998 | 13 |  | BakHapD | F | calf | 120 | 122 | 135 | 141 | 201 | 203 | 170 | 178 | 112 | 112 | 116 | 118 |
| Eau07AI026 | 2007 |  |  | BakHapD | F | cow | 122 | 122 | 135 | 141 | 201 | 203 | 170 | 178 | 112 | 112 | 116 | 118 |
|  |  |  |  |  |  |  |  |  |  |  |  |  |  |  |  |  |  |  |
| Eau96AI047 | 1996 | 11 | 0 | BakHapA | M | calf | 122 | 122 | 122 | 133 | 203 | 203 | 166 | 170 | 108 | 112 | 108 | 114 |
| Eau07AI193 | 2007 |  |  | BakHapA | M | non-calf | 122 | 122 | 122 | 133 | 203 | 203 | 166 | 170 | 0 | 0 | 108 | 114 |
|  |  |  |  |  |  |  |  |  |  |  |  |  |  |  |  |  |  |  |
| Eau98AI066 | 1998 | 11 | 0 | BakHapA | F | calf | 126 | 126 | 133 | 135 | 191 | 203 | 162 | 166 | 116 | 116 | 116 | 118 |
| Eau07AI194 | 2007 |  |  | BakHapA | F | cow | 126 | 126 | 133 | 135 | 191 | 203 | 162 | 166 | 0 | 0 | 116 | 118 |
|  |  |  |  |  |  |  |  |  |  |  |  |  |  |  |  |  |  |  |
| Eau06AI027 | 2006 | 12 | 0 | BakHapA | M | calf | 126 | 136 | 135 | 137 | 189 | 203 | 170 | 178 | 108 | 108 | 112 | 120 |
| Eau07AI104 | 2007 |  |  | BakHapA | M | non-calf | 126 | 136 | 135 | 137 | 189 | 203 | 170 | 178 | 108 | 108 | 112 | 120 |
| **Sample Name** | **Year** | **N_M_** | **N_MM_** | **mtDNA** | **sex** | **DC** | **EV1** | | **EV14** | | **EV37** | | **GATA28** | | **GATA98** | | **GT23** | |
| Eau06AI029 | 2006 | 8 | 0 | BakHapB+ | M | calf | 0 | 0 | 0 | 0 | 191 | 201 | 0 | 0 | 108 | 116 | 116 | 120 |
| Eau07AI233 | 2007 | to | 0 | BakHapB+ | M | non-calf | 134 | 148 | 133 | 135 | 191 | 201 | 170 | 174 | 108 | 116 | 116 | 120 |
| Eau09AI044 | 2009 | 12 |  | BakHapB+ | M | non-calf | 134 | 148 | 133 | 135 | 191 | 201 | 170 | 174 | 108 | 116 | 116 | 120 |
|  |  |  |  |  |  |  |  |  |  |  |  |  |  |  |  |  |  |  |
| Eau06AI037 | 2006 | 13 | 0 | BakHapD | M | calf | 122 | 140 | 129 | 141 | 203 | 203 | 166 | 178 | 116 | 120 | 108 | 108 |
| Eau07AI081 | 2007 |  |  | BakHapD | M | non-calf | 122 | 140 | 129 | 141 | 203 | 203 | 166 | 178 | 116 | 120 | 108 | 108 |
|  |  |  |  |  |  |  |  |  |  |  |  |  |  |  |  |  |  |  |
| Eau07AI196 | 2007 | 9 | 0 | BakHapD | F | calf | 122 | 124 | 0 | 0 | 189 | 203 | 0 | 0 | 0 | 0 | 108 | 116 |
| Eau08AI022 | 2008 |  |  | BakHapD | F | non-calf | 122 | 124 | 135 | 141 | 189 | 203 | 170 | 174 | 104 | 112 | 108 | 116 |
|  |  |  |  |  |  |  |  |  |  |  |  |  |  |  |  |  |  |  |
| Eau07AI025 | 2007 | 13 | 0 | BakHapD | F | calf | 122 | 130 | 133 | 137 | 191 | 201 | 166 | 170 | 112 | 112 | 110 | 114 |
| Eau08AI044 | 2008 |  |  | BakHapD | F | non-calf | 122 | 130 | 133 | 137 | 191 | 201 | 166 | 170 | 112 | 112 | 110 | 114 |
|  |  |  |  |  |  |  |  |  |  |  |  |  |  |  |  |  |  |  |
| Eau06AI018 | 2006 | 10 | 0 | BakHapB+ | M | calf | 140 | 142 | 0 | 0 | 189 | 195 | 0 | 0 | 112 | 112 | 110 | 118 |
| Eau08AI201 | 2008 |  |  | N/A | M | non-calf | 140 | 142 | 0 | 0 | 189 | 195 | 162 | 166 | 112 | 112 | 0 | 0 |
|  |  |  |  |  |  |  |  |  |  |  |  |  |  |  |  |  |  |  |
| Eau06AI134 | 2006 | 13 | 0 | BakHapA | M | calf | 122 | 142 | 135 | 139 | 187 | 203 | 166 | 174 | 108 | 112 | 116 | 120 |
| Eau09AI191 | 2009 |  |  | BakHapA | M | non-calf | 122 | 142 | 135 | 139 | 187 | 203 | 166 | 174 | 108 | 112 | 116 | 120 |
|  |  |  |  |  |  |  |  |  |  |  |  |  |  |  |  |  |  |  |
| Eau07AI078 | 2007 | 9 | 0 | PATMALHAPB | F | calf | 128 | 132 | 129 | 137 | 199 | 201 | 178 | 178 | 104 | 116 | 114 | 116 |
| Eau09AI183 | 2009 |  |  | PATMALHAPB | F | non-calf | 0 | 0 | 129 | 137 | 0 | 0 | 178 | 178 | 0 | 0 | 114 | 116 |
|  |  |  |  |  |  |  |  |  |  |  |  |  |  |  |  |  |  |  |
| Eau08AI079 | 2008 | 13 | 0 | B' | F | calf | 126 | 142 | 133 | 139 | 193 | 195 | 166 | 166 | 108 | 116 | 116 | 120 |
| Eau09AI134 | 2009 |  |  | B' | F | non-calf | 126 | 142 | 133 | 139 | 193 | 195 | 166 | 166 | 108 | 116 | 116 | 120 |
|  |  |  |  |  |  |  |  |  |  |  |  |  |  |  |  |  |  |  |
| Eau07AI199 | 2007 | 9 | 0 | A | F | calf | 126 | 126 | 135 | 137 | 0 | 0 | 166 | 178 | 0 | 0 | 114 | 116 |
| Eau09AI108 | 2009 |  |  | A | F | non-calf | 126 | 126 | 135 | 137 | 195 | 199 | 166 | 178 | 108 | 116 | 114 | 116 |

| **Sample Name** | **RW18** | | **RW31** | | **RW410** | | **RW48a** | | **TR3F4** | | **TR3G1** | | **TR3G2** | | **N_loci_** |
| --- | --- | --- | --- | --- | --- | --- | --- | --- | --- | --- | --- | --- | --- | --- | --- |
| Eau95AI017 | 0 | 0 | 121 | 125 | 210 | 211 | 0 | 0 | 317 | 337 | 238 | 238 | 176 | 184 | 11 |
| Eau96AI017 | 197 | 199 | 121 | 125 | 210 | 211 | 120 | 122 | 317 | 337 | 238 | 238 | 176 | 184 | 13 |
| Eau98AI035 | 197 | 199 | 121 | 125 | 210 | 211 | 120 | 122 | 317 | 337 | 0 | 0 | 176 | 184 | 10 |
| Eau06AI039 | 197 | 199 | 121 | 125 | 210 | 211 | 120 | 122 | 317 | 337 | 238 | 238 | 176 | 184 | 13 |
|  |  |  |  |  |  |  |  |  |  |  |  |  |  |  |  |
| Eau98AI106 | 187 | 195 | 121 | 125 | 209 | 211 | 120 | 122 | 305 | 329 | 206 | 206 | 172 | 176 | 13 |
| Eau07AI026 | 187 | 195 | 0 | 0 | 209 | 211 | 120 | 122 | 305 | 329 | 0 | 0 | 172 | 176 | 11 |
|  |  |  |  |  |  |  |  |  |  |  |  |  |  |  |  |
| Eau96AI047 | 187 | 195 | 123 | 123 | 197 | 197 | 118 | 120 | 301 | 301 | 206 | 238 | 172 | 172 | 13 |
| Eau07AI193 | 0 | 0 | 123 | 123 | 197 | 197 | 118 | 120 | 301 | 301 | 206 | 238 | 172 | 172 | 11 |
|  |  |  |  |  |  |  |  |  |  |  |  |  |  |  |  |
| Eau98AI066 | 193 | 195 | 123 | 125 | 195 | 207 | 118 | 122 | 301 | 317 | 222 | 242 | 172 | 172 | 13 |
| Eau07AI194 | 0 | 0 | 123 | 125 | 195 | 207 | 118 | 122 | 301 | 317 | 222 | 242 | 172 | 172 | 11 |
|  |  |  |  |  |  |  |  |  |  |  |  |  |  |  |  |
| Eau06AI027 | 187 | 199 | 123 | 123 | 207 | 210 | 118 | 122 | 301 | 333 | 0 | 0 | 172 | 172 | 12 |
| Eau07AI104 | 187 | 199 | 123 | 123 | 207 | 210 | 118 | 122 | 301 | 333 | 0 | 0 | 172 | 172 | 12 |
|  |  |  |  |  |  |  |  |  |  |  |  |  |  |  |  |
| Eau06AI029 | 187 | 193 | 117 | 123 | 195 | 211 | 120 | 124 | 301 | 320 | 0 | 0 | 172 | 188 | 9 |
| Eau07AI233 | 187 | 193 | 117 | 123 | 195 | 211 | 120 | 124 | 301 | 320 | 214 | 222 | 172 | 188 | 13 |
| Eau09AI044 | 0 | 0 | 117 | 123 | 195 | 211 | 120 | 124 | 301 | 320 | 214 | 222 | 172 | 188 | 12 |
|  |  |  |  |  |  |  |  |  |  |  |  |  |  |  |  |
| Eau06AI037 | 193 | 213 | 121 | 123 | 211 | 211 | 120 | 124 | 305 | 305 | 222 | 238 | 168 | 180 | 13 |
| Eau07AI081 | 193 | 213 | 121 | 123 | 211 | 211 | 120 | 124 | 305 | 305 | 222 | 238 | 168 | 180 | 13 |
|  |  |  |  |  |  |  |  |  |  |  |  |  |  |  |  |
| Eau07AI196 | 0 | 0 | 125 | 125 | 197 | 211 | 118 | 120 | 313 | 329 | 238 | 238 | 172 | 176 | 9 |
| Eau08AI022 | 189 | 195 | 125 | 125 | 197 | 211 | 118 | 120 | 313 | 329 | 238 | 238 | 172 | 176 | 13 |
|  |  |  |  |  |  |  |  |  |  |  |  |  |  |  |  |
| **Sample Name** | **RW18** | | **RW31** | | **RW410** | | **RW48** | | **TR3F4** | | **TR3G1** | | **TR3G2** | | **N loci** |
| Eau07AI025 | 189 | 199 | 121 | 121 | 205 | 207 | 108 | 108 | 305 | 308 | 206 | 242 | 172 | 172 | 13 |
| Eau08AI044 | 189 | 199 | 121 | 121 | 205 | 207 | 108 | 108 | 305 | 308 | 206 | 242 | 172 | 172 | 13 |
|  |  |  |  |  |  |  |  |  |  |  |  |  |  |  |  |
| Eau06AI018 | 187 | 231 | 123 | 125 | 195 | 195 | 118 | 122 | 305 | 317 | 206 | 238 | 176 | 184 | 11 |
| Eau08AI201 | 187 | 231 | 123 | 125 | 195 | 195 | 118 | 122 | 305 | 317 | 206 | 238 | 176 | 184 | 11 |
|  |  |  |  |  |  |  |  |  |  |  |  |  |  |  |  |
| Eau06AI134 | 193 | 225 | 123 | 125 | 203 | 209 | 118 | 118 | 301 | 305 | 234 | 238 | 168 | 172 | 13 |
| Eau09AI191 | 193 | 225 | 123 | 125 | 203 | 209 | 118 | 118 | 301 | 305 | 234 | 238 | 168 | 172 | 13 |
|  |  |  |  |  |  |  |  |  |  |  |  |  |  |  |  |
| Eau07AI078 | 193 | 219 | 117 | 119 | 199 | 199 | 118 | 126 | 301 | 317 | 206 | 222 | 168 | 180 | 13 |
| Eau09AI183 | 193 | 219 | 117 | 119 | 199 | 199 | 118 | 126 | 301 | 317 | 0 | 0 | 168 | 180 | 9 |
|  |  |  |  |  |  |  |  |  |  |  |  |  |  |  |  |
| Eau08AI079 | 193 | 217 | 123 | 125 | 191 | 195 | 106 | 122 | 305 | 333 | 222 | 238 | 172 | 172 | 13 |
| Eau09AI134 | 193 | 217 | 123 | 125 | 191 | 195 | 106 | 122 | 305 | 333 | 222 | 238 | 172 | 172 | 13 |
|  |  |  |  |  |  |  |  |  |  |  |  |  |  |  |  |
| Eau07AI199 | 0 | 0 | 123 | 131 | 205 | 209 | 118 | 120 | 301 | 313 | 0 | 0 | 176 | 184 | 9 |
| Eau09AI108 | 195 | 195 | 123 | 131 | 205 | 209 | 118 | 120 | 301 | 313 | 202 | 234 | 176 | 184 | 13 |
